# BiLSTM-I: A Deep Learning-Based Long Interval Gap-Filling Method for Meteorological Observation Data

**DOI:** 10.3390/ijerph181910321

**Published:** 2021-09-30

**Authors:** Chuanjie Xie, Chong Huang, Deqiang Zhang, Wei He

**Affiliations:** 1 State Key Laboratory of Resources and Environmental Information System, Institute of Geographic Sciences and Natural Resources Research, Chinese Academy of Sciences, Beijing 100101, China; xiecj@lreis.ac.cn (C.X.); hew.20s@igsnrr.ac.cn (W.H.); 2 Key Laboratory of Plant Resources Conservation and Sustainable Utilization, South China Botanical Garden, Chinese Academy of Sciences, Guangzhou 510650, China; zhangdeq@scbg.ac.cn

**Keywords:** time series, data imputation, deep learning, meteorological observation data

## Abstract

Complete and high-resolution temperature observation data are important input parameters for agrometeorological disaster monitoring and ecosystem modelling. Due to the limitation of field meteorological observation conditions, observation data are commonly missing, and an appropriate data imputation method is necessary in meteorological data applications. In this paper, we focus on filling long gaps in meteorological observation data at field sites. A deep learning-based model, BiLSTM-I, is proposed to impute missing half-hourly temperature observations with high accuracy by considering temperature observations obtained manually at a low frequency. An encoder-decoder structure is adopted by BiLSTM-I, which is conducive to fully learning the potential distribution pattern of data. In addition, the BiLSTM-I model error function incorporates the difference between the final estimates and true observations. Therefore, the error function evaluates the imputation results more directly, and the model convergence error and the imputation accuracy are directly related, thus ensuring that the imputation error can be minimized at the time the model converges. The experimental analysis results show that the BiLSTM-I model designed in this paper is superior to other methods. For a test set with a time interval gap of 30 days, or a time interval gap of 60 days, the root mean square errors (RMSEs) remain stable, indicating the model’s excellent generalization ability for different missing value gaps. Although the model is only applied to temperature data imputation in this study, it also has the potential to be applied to other meteorological dataset-filling scenarios.

## 1. Introduction

Temperature is a very important variable for agricultural and ecosystem studies, and it is an essential input in agricultural crop growth simulations, agrometeorological disaster monitoring, and ecosystem simulations [1,2]. As agricultural and ecological simulations have improved, the resolution requirements for temperature data have increased; notably, high-resolution data are needed in wind monitoring in dry and hot areas, agrometeorological hazard assessments, and simulations of carbon emissions from forest block ecosystems [3,4]. Temperature observations are usually obtained from field meteorological stations, and the data observed at small weather stations commonly have gaps due to equipment failure, harsh environmental conditions or operational errors [5]. The imputation or completion of missing data is an essential preprocessing task before temperature observation data are applied.

There are various methods for data imputation, and they can be classified into three main categories: deterministic model-based methods, statistical model-based methods and machine learning methods [6]. Deterministic models are based on observed values and can interpolate missing values using deterministic mathematical methods, such as the overall average, nearest neighbour, polynomial, and spline function interpolation methods for unobserved values [7,8]. Imputation methods based on statistical models fully consider the observation error and the error generated in the imputation process to reduce the error in the imputation results through optimization. The regression method is representative of such methods, and it obtains mathematical expressions of observed values through regression and then interpolates the missing values using mathematical expressions [9]; various time series regression imputation methods have been widely used [10,11]. An imputation method that combined a Kalman filter and time series regression analysis performed well in the imputation of missing values in single-factor time series [5,12]. The accuracy of time series data imputation depends on the closeness between the time series representation model in the algorithm and the ‘real’ model. Traditional methods of time series data imputation generally assume a predefined model structure for time series data. The fitness of the predefined model has a great impact on the accuracy of data imputation.

Alternatively, machine learning involves learning the potential distribution of data from the acquired observations and interpolating missing values with a model established after learning. Data imputation methods based on traditional machine learning include those based on principal component analysis, low-rank matrix decomposition, kernel methods [13,14,15], and combined data imputation methods [16]. Modern machine learning imputation methods can be applied in data imputation by applying deep learning techniques; this approach provides a rich and diverse network structure [17,18] and is suitable for univariate or multivariate time-series imputation [19,20]. The active learning process may obtain a better representation model much closer to the real data structure, thus obtaining a higher data imputation accuracy.

Meteorological observations are typical time-series data. Time-series imputation methods, such as mean imputation, stochastic regression imputation are generally available for filling in missing values in meteorological observations. Although methods of imputing missing values in time series are abundant, research on how to use low-frequency manually acquired observations to fill the long time interval gaps in high-frequency machine-based observations is lacking [21]. Considering a common situation, an ecological station collects the temperature data using an automatic weather station in the field, and manual temperature observation is also employed at the same time. The temporal frequency of the observation product of the automatic meteorological data output is high, for example, one record per 30 min, with 48 observation records per day; manual temperature observations are obtained in the morning, at midday and in the evening three times per day, resulting in only three manual observation records. Although the automatic meteorological data are superior to manual observations on recording frequency, they are greatly affected by occasional factors, such as the bad weather, the problem of facilities, etc., which might easily lead to long-time-interval data loss.

In this study, we proposed a new deep learning-based model BiLSTM-I to obtain complete half-hourly-frequency temperature observation datasets based on daily manually observed temperature data. We detailed the model structure. Taking a forest ecology station in Guangzhou, China as an example, we elaborated the application of our model to fill the long time interval gap of automatic temperature observation data. Moreover, we compared our results with other classical methods for missing data imputation to highlight the efficiency of our model.

## 2. Materials and Methods

### 2.1. Meteorological Temperature Observation Data

In this study, meteorological temperature observation data from the National Field Scientific Observation and Research Station of the Dinghushan Forest Ecosystem (23.18° N, 112.53° E) in Guangzhou, China, were used. This ecosystem observatory has performed comparative temperature observation experiments with both manual observations and automatic meteorological machine-based observations and has a long record of temperature observation data. The following Table 1 lists the manual temperature observation data and automatic machine observation data used in this study.

**Figure 1:**
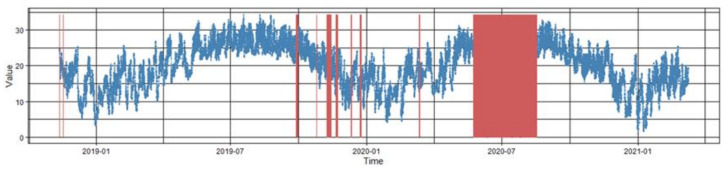
Distribution of missing values in half-hourly temperature observation data.

Since the Dinghushan Ecological Station is located in the mountainous region of southern China, the automatic observation equipment is susceptible to external effects, which may lead to missing observation records for long periods of time, especially in the thunderstorm season. Figure 1 shows the distribution of missing meteorological observation data; notably, there were missing temperature observations for more than 2 months around July 2020.

This article focuses on the imputation of missing machine temperature observations for more than 2 months around July 2020 using manual observations obtained three times a day. Since linear correlations can be easily established between manual and machine observations, the core objective of the data imputation problem is determining how to apply low-frequency manually obtained temperature observations to fill long-time-interval gaps in data sets of high-frequency automatic machine temperature observations.

### 2.2. Baseline Methods

#### 2.2.1. Time Series Data Imputation with Kalman Smoothing

Kalman smoothing has the same mathematical basis as the widely used Kalman filter, both of which involve estimating unobservable system states from observable data. The Kalman filter method has linear and non-linear forms, and the basic linear Kalman filter equation is used in this case. The evolution of the system state space can be expressed as Equations (1) and (2) [22,23].

(1) $$a_{t}=T_{t}a_{t-1}+R_{t}\eta_{t}\;\;\;\;\;\;\;\eta_{t}\sim N\left(0,Q_{t}\right)\;\;$$

(2) $$y_{t}=Z_{t}a_{t}+\varepsilon_{t}\;\;\;\;\;\;\;\;\;\;\;\;\;\;\;\varepsilon_{t}\sim N\left(0,H_{t}\right)\;$$

where $a_{t}$ is an unobservable system state, $T_{t}$ is the state transfer matrix, $R_{t}$ is the system noise-driven matrix, $y_{t}\;$ is the observed data, and *Z_t_* is the observation matrix. $\eta_{t}$ and $\;\varepsilon_{t}$ denote the white noise of the state transform process and measurement, and they are independent of each other.

Following the Kalman filter and smoothing methods, the best estimate of the system state ${\tilde{a}}_{t|n}$ can be obtained assuming an observation set $Y_{t}=\left(y_{1},y_{2},\cdots ,y_{n}\right)$ with n samples; the corresponding estimation error covariance matrix is $P_{t|n}={\left(a_{t}-{\tilde{a}}_{t|n}\right)}^{T}\left(a_{t}-{\tilde{a}}_{t|n}\right)$. Kalman filtering provides an estimation of the current system state from observations, and smoothing yields an estimation of the past system state; the best estimation processes for specific system states have been described in many studies [24].

Kalman smoothing, which uses all temperature observations available before and after the missing value window, provides the best estimation of the state at any moment in a previous observation period and can be used to obtain valid estimates of the missing temperature observations. To apply Kalman smoothing, a state space model, such as that in Equations (1) and (2), is required. These equations include the matrices $T_{t}$, $Z_{t}$, and $R_{t}$. The state equations are developed using a structured time series model and a time series regression model.

(1) Structured time series model (Kalman-S)

The basic structured (BSM: basic structured model) time series model is used here, and the basic BSM formulas are as follows Equations (4)–(6) [25,26]:

(3) $$y_{t}=\mu_{t}+\gamma_{t}+\varepsilon_{t}$$

(4) $$\mu_{t}=\mu_{t-1}+\beta_{t}+\eta_{t}$$

(5) $$\beta_{t}=\beta_{t-1}+\xi_{t}$$

(6) $$\gamma_{t}=-\overset{s-1}{\underset{j=1}{\sum }}\gamma_{t-j}+\omega_{t}$$

In the above equation set, Equation (3) is the observed equation for time series $y_{t}$, where $\mu_{t}\;$ is the trend component and is linearly approximated by Equations (4) and (5); $\gamma_{t}$ is the seasonal component of the time series, which is defined by Equation (6); $\varepsilon_{t},\;\eta_{t},\;\xi_{t}\;\mathrm{and}\;\omega_{t}$ in the above equations are the mean zero and variance of $\delta_{\varepsilon }^{2},\;\delta_{\eta }^{2},\;\delta_{\xi }^{2}\;\mathrm{and}\;\delta_{\omega }^{2}$ for mutually independent noise, respectively; *s* in Equation (6) is the number of seasonal cycles of the time series in a year.

By transformation, the BSM equations can be transformed into state model expression form. For simplicity, we can set *s* to 4 and obtain Equations (7) and (8):

(7) $$a_{t}\equiv \left[\begin{array}{c} \mu_{t}\\ \beta_{t}\\ \gamma_{t}\\ \gamma_{t-1}\\ \gamma_{t-2} \end{array}\right]=\left[\begin{array}{ccccc} 1 & 1 & 0 & 0 & 0\\ 0 & 1 & 0 & 0 & 0\\ 0 & 0 & -1 & -1 & -1\\ 0 & 0 & 1 & 0 & 0\\ 0 & 0 & 0 & 1 & 0 \end{array}\right]\cdot \left[\begin{array}{c} \mu_{t-1}\\ \beta_{t-1}\\ \gamma_{t-1}\\ \gamma_{t-2}\\ \gamma_{t-3} \end{array}\right]+\left[\begin{array}{ccc} 1 & 0 & 0\\ 0 & 1 & 0\\ 0 & 0 & 1\\ 0 & 0 & 0\\ 0 & 0 & 0 \end{array}\right]\cdot \left[\begin{array}{c} \eta_{t}\\ \xi_{t}\\ \omega_{t} \end{array}\right]$$

(8) $$y_{t}=\;\left[\begin{array}{ccccc} 1 & 0 & 1 & 0 & 0 \end{array}\right]a_{t}+\varepsilon_{t}$$

By comparing Equations (7) and (8) with the Kalman smoothing state Equations (1) and (2) above, the expressions required to transform the BSM equations into a state model can be obtained.

(2) ARIMA-based state space model (Kalman-A)

The differential integrated moving average autoregressive model (ARIMA: autoregressive integrated moving average) is a widely used time-series forecasting method and is also widely used in single-factor time-series analysis [27]. The ARIMA-based state model has been applied to problems involving traffic state forecasting and missing value imputation for time series [5,28]. Compared with ARMA (autoregressive moving average model), ARIMA first enhances the stability of observed time series through difference operations, and ARMA is then used to model the time series. The mathematical expressions of both are consistent, and ARIMA is used below in the introduction of the state model establishment process [5,29,30].

#### 2.2.2. BRITS-I Time Series Imputation Method Based on Deep Learning

Deep learning is an effective method for the imputation of time series data [31], for example, a recurrent neural network (RNN) was used to impute missing values in a smooth fashion [10]. The BRITS-I method [32] uses RNN to predict the missing values directly in a recurrent dynamical system based on the observed data. As a simpler case of BRITS-I, RITS-I employs a unidirectional recurrent dynamical system, in which the missing value in the time series can be derived by its predecessors with a fixed arbitrary function. The algorithm contained a recurrent component implemented by a RNN and a regression component represented by a fully-connected network. A standard recurrent network [17] can be represented as Equation (9):

(9) $$h_{t}=\sigma \left(W_{h}h_{t-1}+U_{h}x_{t}+b_{h}\right)\;$$

where σ is the sigmoid function, $W_{h}$, $U_{h}$ and $b_{h}$ are parameters, and $h_{t}$ is the hidden state of previous time steps.

Considering that the time series may be irregularly sampled, a temporal decay factor $\gamma_{t}$ was introduced in RITS-I, which represents the missing patterns in the time series Equation (10).

(10) $$\gamma_{t}=exp\;\left\{-max\left(0,W_{\gamma }\delta_{t}+b_{\gamma }\right)\right\}\;$$

In a unidirectional recurrent dynamical system, errors of estimated missing values are delayed until the presence of the next observation. To alleviate the issue, BRITS-I utilized the bidirectional recurrent dynamics on the given time series, i.e., besides the forward direction, each value in time series can be also derived from the backward direction by another fixed arbitrary function [32].

### 2.3. BiLSTM-I Model Development

Several studies showed that neural networks with sequence-to-sequence (Seq2Seq) structures can efficiently fill gaps in time series [32,33]. However, deep learning models, with different structures, designs and optimization objective functions, can exhibit large performance differences when solving similar problems. The imputation model BiLSTM-I proposed in this paper designed an encoder-decoder deep learning architecture, and an optimization objective error function, to obtain high accuracy in long interval gap filling for time-series meteorological observation data.

#### 2.3.1. Basic Definition

The temperature displays periodicity on the scale of days, and it is natural to divide long time series of half-hourly temperature observations over days into a segmented series of 48 observations per day. To focus on the imputation of missing values over long time intervals, occasional or short-term gaps in the time series are first interpolated using the Kalman smoothing method described above. The temperature time series thus included two segment types: daily segments without missing values, denoted as $\;d_{full}^{j}$, and daily segments containing observations in the morning, afternoon, and evening, denoted as $d_{miss}^{j}$. The time-segmented series can be expressed as Equation (11):

(11) $$\{\;d_{full}^{1},\ldots ,\;d_{full}^{i},\;d_{miss}^{i+1},\ldots ,\;d_{miss}^{i+m},\;d_{full}^{i+m+1},\ldots ,\;d_{full}^{n}\}$$

In this study, we used temperature time series of two years. Therefore, sequence (11) is a temperature time series of length 730 days (n). The missing value window width of m was set to 30 and 60 days, respectively. For the half-hourly temperature observation, sequence (11) represents a temperature observation data sequence of length 35,040 (L) with 1440 and 2880 missing values expressed in the form of daily segmentation.

To represent the positions of the missing values in the time series (11), a mask time series {$m_{t}^{i}$} of corresponding length *L* is constructed using Equation (12) for the half-hourly sampled temperature time series {$T_{t}^{i}$} of length *L*, where:

(12) $$m_{t}^{i}=\left\{\begin{array}{c} 0\;,As\;T_{t}^{i}\;Unobserved\\ 1\;\;\;\;,\;\;\;\;\;\;\;\;\;\;\;\;\;\;else \end{array}\right.$$

Now, the half-hourly mask sequence of length *L* is segmented in days, the mask without missing values is segmented daily as $M_{full}^{j}$, and the mask containing only three observations in the morning, afternoon and evening is segmented daily as $M_{miss}^{j}$; therefore, the mask sequence corresponding to (11) segmented in days can be constructed as (13):

(13) $$\{\;M_{full}^{1},\ldots ,\;M_{full}^{i},\;M_{miss}^{i+1},\ldots ,\;M_{miss}^{i+m},\;M_{full}^{i+m+1},\ldots ,\;M_{full}^{n}\}$$

#### 2.3.2. Rolling Window Sampling

A rolling window approach is used to construct a sample set for deep learning model training based on the time series segmented by day. For the time interval gap length of m days, the length of the rolling window needs to be constructed to be greater than *m*, and observations of length *s* (days) are kept at each end of *m* so that the rolling window length *w* is *m + 2 × s* days. The training samples are constructed by adapting the Seq2Seq training method to the training input sample of length *w*; the temperature observation sequence (14) is:

(14) $$\left\{\;d_{full}^{1},\ldots ,\;d_{full}^{s},\;d_{miss}^{s+1},\ldots ,\;d_{miss}^{s+m},\;d_{full}^{s+m+1},\ldots ,\;d_{full}^{w}\right\}$$

The following time series output (15) can be obtained from the training process:

(15) $$\left\{\;d_{full}^{1},\ldots ,\;d_{full}^{s},\;{\hat{d}}_{impt}^{s+1},\ldots ,\;{\hat{d}}_{impt}^{s+m},\;d_{full}^{s+m+1},\ldots ,\;d_{full}^{w}\right\}$$

In sequence (15), ${\hat{d}}_{impt}^{j}$ is the complete segment of the observed temperature values for each half-hour in a day after the imputation of missing values. In constructing the training samples, a mask sequence of length *w* (days) is constructed as another input to the training samples according to the observation values corresponding to the mask sequence on the order of days.

The training sample is constructed based on the temperature observation sequence without missing values, and the pattern of missing observations in the sample is consistent with the actual situation; only three observations per day in the morning, afternoon, and evening are considered. Table 2 gives an example of a day of temperature data with missing values in the training sample and the corresponding mask.

#### 2.3.3. Design of Deep Learning Models

Typical Seq2Seq-based deep learning models for the imputation of time series data are SSIM and BRITS-I [34,35]. In this paper, the advantages of these models are utilized, an encoder-decoder deep learning architecture is adopted, and the structure of the designed deep learning model is shown in the following figure. For convenience, the above input sequence (13) is denoted as x = {x_1_, x_2_..., x_n_}, the output sequence (14) is denoted as y = {y_1_, y_2_..., y_n_}, and the mask sequence (12) is denoted as m = {m_1_, m_2_..., m_n_}.

(1) Encoder

**Figure 2:**
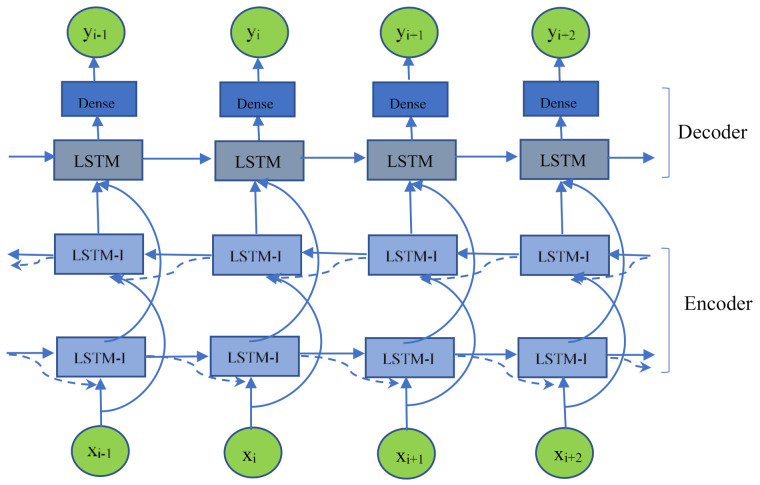
Structure of the imputation neural network for missing temperature values.

As shown in Figure 2, the basic structure of the encoding part of the deep learning structure is based on LSTM-I. The core of the LSTM-I unit is an LSTM neural network unit. The recurrent neural network unit directly adopts a long short-term memory (LSTM) unit, which is a special kind of RNN, to solve the gradient disappearance and gradient explosion problems during the training of long sequences. In addition, the missing value part of the temperature observation set in this paper, with 48 half-hourly temperature values daily, contains only 3 observations, so the variable $y_{t}$ in Equation (10) for missing value intervals is not used.

The LSTM structure and mathematical description can be found in reference [36], and the LSTM is reduced to the form of a simple operator in the following definition. The following mathematical description of the LSTM-I unit process is given:

(16) $${\tilde{x}}_{t}\;=\;W_{x}h_{t-1}+b_{x}$$

(17) $$x_{t}^{c}=x_{t}⊙m_{t}\;+\;(1-m_{t})⊙{\tilde{x}}_{t}$$

(18) $$h_{t}=\mathrm{LSTM}(x_{t}^{c},h_{t-1})$$

(19) $$l_{t}=\;<m_{t},\;£(x_{t},\;{\tilde{x}}_{t})>$$

Equation (16) transforms the hidden state *h_t−1_* of the previous LSTM cell into the estimated vector $\tilde{x}$*_t_*, where *W_x_* and *b_x_* are model parameters. Equation (17) replaces a missing value in the input vector *x_t_* with the value corresponding to the estimated vector $\tilde{x}$*_t_* by applying the mask vector *m_t_*. Equation (18) generates the predicted state *h_t_* through the LSTM network cell with $x_{t}^{c}$ and the hidden state *h_t−1_* as inputs. Equation (19) is the estimation error of the LSTM-I cell as the cumulative absolute difference between the observed and estimated values at the location of a missing value.

The encoding part of the neural network in the figure consists of a bidirectional LSTM-I neural network. An LSTM-I reads the input from the beginning to the end of the time series and generates a forward hidden-state vector sequence $\vec{h}=\{\vec{h_{1}},\vec{h_{2}},\ldots ,\vec{h_{n}}\}$; the other LSTM-I reads the input backwards from the end of the time series to the beginning and produces a backward hidden-state sequence $\overset{↼}{h}=\{\overset{↼}{h_{1}},\overset{↼}{h_{2}},\ldots ,\overset{↼}{h_{n}}\}$. The forward and backward hidden-state sequences are stitched together to form the encoded output *h* = {*h_1_, h_2_,…, h_n_*} of the encoding layer, where the vector *h_i_* is *h_i_* = {$\vec{h}$*_i_,*$\;\overset{↼}{h}$*_i_*}.

The bidirectional LSTM-I encoding network error includes both forward and backward estimation errors.

(2) Decoder

The decoding layer receives the encoded output sequence h and produces the resulting time series sequence y. The neural network decoding process is mathematically described as Equations (20)–(22):

s_t_ = LSTM(*h_t_*, s_t−1_) (20)

y_t_ = W_y_s_t_ + b_y_ (21)

(22) $$l_{y}=\;<m_{t},\;£(x_{t},\;y_{t})>$$

As in Equation (20), the bottom of the decoding layer is a standard LSTM network that synthesizes the encoded output sequence h to produce an output state sequence *s* = {*s_1_, s_2_..., s_n_*} containing valuable information. As in Equation (21), since the temperature values are continuous, a linear fully connected layer is used at the top of the decoding layer to output the imputation-based sequence *y*. Equation (22) gives the error of imputation results for the decoding layer, and this value is the cumulative absolute difference between the observed and interpolated values at the location of a missing value.

The error function of the entire neural network consists of three components Equation (23):

(23) $$l_{t}=l_{t}^{f}+l_{t}^{b}+l_{y}$$

In Equation (23), $l_{t}^{f}$ is the estimation error of the forward LSTM-I encoding layer, and $l_{t}^{b}$ is the estimation error of the backward LSTM-I encoding layer.

### 2.4. Accuracy Evaluation

In this paper, several metrics are used to evaluate the performance of different data imputation methods, and the values of the evaluation metrics are calculated based on the test sample set. These metrics include the root mean square error (RMSE) (Equation (24)), mean absolute error (MAE) (Equation (25)), mean relative error (MRE) (Equation (26)) and Pearson correlation coefficient (PCC) (Equation (27)), which are defined as follows.

(24) $$RMSE=\sqrt{\frac{1}{n}\sum_{i=1}^{n}{\left(x_{i}-y_{i}\right)}^{2}}$$

(25) $$MAE=\frac{1}{n}\overset{n}{\underset{i=1}{\sum }}\left|x_{i}-y_{i}\right|$$

(26) $$MRE=\frac{1}{n}\overset{n}{\underset{i=1}{\sum }}\left|\frac{x_{i}-y_{i}}{x_{i}}\right|$$

(27) $$PCC=\frac{\sum_{i=1}^{n}(x_{i}-\bar{x})\left(y_{i}-\bar{y}\right)}{\sum_{i=1}^{n}{\left(x_{i}-\bar{x}\right)}^{2}\sum_{i=1}^{n}{\left(y_{i}-\bar{y}\right)}^{2}}$$

In the above index formulas, *x_i_* is an actual missing observation in the constructed test sample set, and *y_i_* is the interpolated result at the location of the missing value. In (27), $\bar{x}$ is the overall average of the actual observed value at the location of a missing value in the sample, and $\bar{y}$ is the overall average of the interpolated result at the location of a missing value in the formula; this value is then used to calculate the *PCC*.

## 3. Results and Discussion

**Figure 3:**
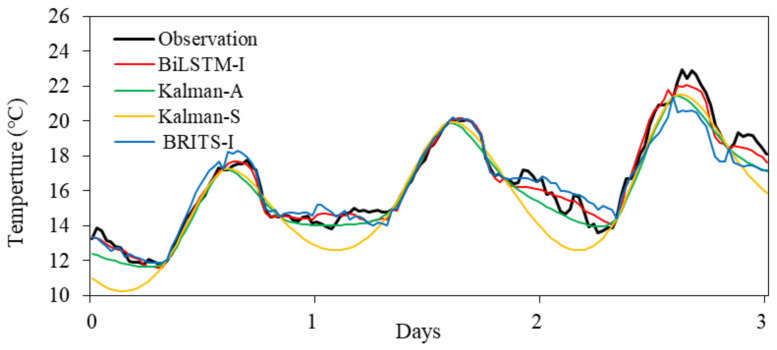
Comparison the imputation results of different methods with the observation data. Data for three days were randomly selected.

The model implementation is based on the open source machine learning framework PyTorch (https://pytorch.org/ accessed on 20 september 2021). The training set is constructed with the observations on the left side of the missing value window for July 2020, and the test set is constructed with the observations on the right side. The deep learning imputation method includes the construction of two training samples: one with a time interval gap of 30 days and another with a time interval gap of 60 days. The acquired observations on both sides of the gap span 14 days. To distinguish these two training samples, the length of the missing value gap is used as the suffix of the corresponding deep learning imputation method below. The imputation results are shown in Figure 3, and the accuracy assessment results of various imputation methods are summarized in Table 3.

A comparison of BRITS-I, the Kalman method and BiLSTM-I from Table 3 indicates that the BiLSTM-I deep learning-based imputation method developed in this paper performs best among all the methods involved. The Kalman imputation methods are better than BRITS-I. For the Kalman imputation methods, the imputation method based on the ARIMA state model yields better RMSE accuracy than Kalman-Struct. Additionally, the BRITS-I deep learning time-series imputation method is associated with the lowest accuracy (Figure 3).

### 3.1. BiLSTM-I vs. BRITS-I

Both BiLSTM-I and BRITS-I methods adopt the architecture of deep learning. According to Table 3, the accuracy of the BiLSTM-I model designed in this paper is higher than that of the BRITS-I model. There are two main differences between the BiLSTM-I model and the BRITS-I model. First, from the perspective of the model structure, BiLSTM-I adopts an encoder-decoder structure, and BRITS-I is equivalent to only the encoder part of the BiLSTM-I model. Such a model structure of BiLSTM-I is conducive to fully learning the potential distribution pattern of data, which can yield a high data imputation accuracy. Second, there is a difference in the model error function. The error functions of BiLSTM-I and BRITS-I consist of three parts [32]; the first two parts are the same, and the third part of the BiLSTM-I model error function involves the difference between the final estimates and true observations; therefore, the BiLSTM-I model error function evaluates the imputation results more directly, and the model convergence error and the imputation accuracy are directly related, thus ensuring that the imputation error can be minimized at the time the model converges.

### 3.2. BiLSTM-I vs. Kalman Smoothing

The observations on both sides of the missing value window around July 2020 are selected, and the time series decomposition equation (BSM) or ARIMA equation of state is obtained through training to establish Kalman smoothing imputation models. The accuracy of the Kalman smoothing imputation method mainly depends on whether the state equations accurately represent the time series characteristics [37]. The Kalman-S assumes that the trend and seasonal components of the time series can be fitted by the basic linear equation; the Kalman-A fits the differenced time series by establishing a regression equation. By comparison, the BiLSTM-I deep learning method does not make any assumptions about the expression form of the time series, and automatically learns the exact expression form of the time series by repeatedly training the data set to reduce the fitting errors. From the test results, the BiLSTM-I method is more likely to obtain the accurate representation of the time series than the BSM- or ARIMA-based Kalman methods, and thus obtains a higher accuracy of data interpolation.

### 3.3. The Generalization Ability of BiLSTM-I

**Figure 4:**
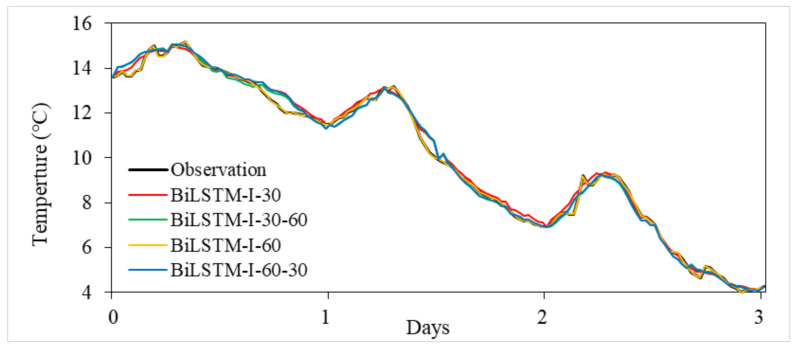
BiLSTM-I model results with 30- and 60-day gaps.

The generalization ability of a model is very important in the application of deep learning methods, and the generalization ability in this paper is reflected in whether the imputation accuracy of the model is consistent for different time interval gaps. In Table 3, the missing value gaps assessed with the BiLSTM-I model are 30 days and 60 days, and the testing accuracy is basically the same for both cases, which indicates a good generalization ability. To further test this ability, we filled a time series of temperature observations with a time interval gap of 30 days by a model trained on a 60-day gap, and vice versa. The model results are shown in Figure 4, and Table 4 shows the accuracy statistics for the results of the imputation methods for these two cases. As shown in Table 4, the indicators of model accuracy are very stable for both cases, which indicates that the BiLSTM-I deep learning model has excellent generalization ability for different missing value gaps.

## 4. Summary

In this paper, a deep learning-based long interval gap-filling model, BiLSTM-I, was proposed for meteorological data imputation. The method addresses the practical problem of using the Seq2Seq-based deep learning technique to obtain complete high-precision, half-hourly frequency temperature observation data based on daily low-frequency temperature observations obtained manually. The experimental analysis results show that the BiLSTM-I designed in this paper outperforms other imputation methods, such as the Kalman smoothing method, or the BRITS-I deep learning method. In addition, BiLSTM-I shows great generalization ability to different missing value gaps. The RMSE for a test set with a missing value gap of 30 days is 0.47, while the RMSE for a test set with a missing value gap of 60 days is 0.49. The model not only meets the high-precision temperature data imputation requirements, but also has the potential to be applied to other meteorological dataset filling scenarios.

## Figures and Tables

**Table 1:** Information table of temperature observation data set.

Dataset	Frequency	Time	Missing Values
Dataset 1	8 am daily	2018/11/13–2020/2/10	None
Dataset 2	2 pm daily	2018/11/13–2020/2/10	None
Dataset 3	8 pm daily	2018/11/13–2020/2/10	None
Dataset 4	Every half hour	2018/11/13–2020/2/10	Short time interval gaps and one long time interval gap

(Datasets 1–3 include manual observation data, and Dataset 4 includes automatic observation data).

**Table 2:** Example of data for a day within the window of missing values in the sample series.

Time	Temperature Value	Mask
1	Na	0
2	Na	0
…	…	…
17	17.14	1
18	Na	0
…	…	…
27	Na	0
28	21.78	1
29	Na	0
…	…	…
39	Na	0
40	19.86	1
41	Na	0
…	…	…
47	Na	0
48	Na	0

The first column of the table is the time sequence number in half hours, starting from 0:00, and the middle column is the temperature value (in Celsius). Only three valid observations are available (morning, midday and evening); the third column is the missing position mask for temperature observations.

**Table 3:** Statistical table of the results of the time series imputation methods.

Method	RMSE (°C)	MAE (°C)	MRE	PCC
BiLSTM-I-60	0.4929	0.3319	0.0173	0.9963
BiLSTM-I-30	0.4686	0.3215	0.0170	0.9968
BRITS-I	1.3959	1.0300	0.0537	0.9697
Kalman-Struct	1.1742	0.8449	0.0472	0.9873
Kalman-ARIMA	0.7514	0.5469	0.0306	0.9934

Mean square error (RMSE); mean absolute error (MAE); mean relative error (MRE); pearson correlation coefficient (PCC).

**Table 4:** Statistics for the imputation accuracy of the BiLSTM-I model applied to missing values over 30- and 60-day gaps.

Missing Value Gap	Model	RMSE	MAE	MRE	PCC
30 Days	BiLSTM-I-30	0.4686	0.3215	0.0170	0.9968
	BiLSTM-I-60	0.4865	0.3326	0.0176	0.9966
60 Days	BiLSTM-I-60	0.4929	0.3319	0.0173	0.9963
	BiLSTM-I-30	0.4834	0.3293	0.0172	0.9964

## Data Availability

Restrictions apply to the availability of these data. Data was obtained from the National Field Scientific Observation and Research Station of Dinghushan Forest Ecosystem and are available the corresponding author with the permission of the National Field Scientific Observation and Research Station of Dinghushan Forest Ecosystem.
